# Predictive factors for responders to tolvaptan in fluid management after cardiovascular surgery

**DOI:** 10.1007/s11748-016-0712-6

**Published:** 2016-09-19

**Authors:** Takashi Kido, Hiroyuki Nishi, Koichi Toda, Takayoshi Ueno, Toru Kuratani, Masayuki Sakaki, Toshiki Takahashi, Yoshiki Sawa

**Affiliations:** 1 Department of Cardiovascular Surgery, Osaka University Graduate School of Medicine, 2-2, Yamada-Oka, Suita, Osaka, 565-0871 Japan; 2 Department of Cardiovascular Surgery, Osaka Police Hospital, 10-31, Kitayama-cho, Tennoji-ku, Osaka, 543-0035 Japan; 3 Department of Cardiovascular Surgery, Osaka National Hospital, 2-14, Hoenzaka, Chuo-ku, Osaka, 540-0006 Japan

**Keywords:** Cardiovascular surgery, Postoperative management, Diuretics, Tolvaptan

## Abstract

**Objective:**

To identify the predictive factors for responders to tolvaptan, a novel vasopressin type 2 receptor antagonist for fluid management after cardiovascular surgery.

**Methods:**

Between January 2013 and May 2014, 113 patients were treated with tolvaptan for fluid management after cardiovascular surgery. As indicators for the effects of tolvaptan, change in bodyweight during the tolvaptan administration period and correlations with perioperative factors were assessed. Thirty-one patients were administered tolvaptan at the first day after surgery (early tolvaptan group). In this group, urine volume during the 6 h before the initial tolvaptan administration was compared with that at 6 h after administration.

**Results:**

For all the patients, the change in bodyweight during the tolvaptan administration period significantly correlated with pre-operative serum creatinine (*r* = 0.19, *p* = 0.04) and albumin levels before tolvaptan administration (*r* = −0.29, *p* = 0.002). In the early tolvaptan group, the ratio of urine volume at 6 h before and 6 h after the initial tolvaptan administration significantly correlated with the pre-operative serum creatinine level (*r* = 0.43, *p* = 0.02), the serum albumin level before tolvaptan administration (*r* = −0.50, *p* = 0.004), and change in bodyweight (*r* = 0.38, *p* = 0.03).

**Conclusions:**

In patients undergoing cardiovascular surgery, deteriorating renal function, increased bodyweight, and hypoalbuminemia were found to be predictive factors for responders to tolvaptan for postoperative fluid management.

## Introduction

Fluid and volume therapy is an important factor in the management of patients after cardiovascular surgery. Currently available options for treating fluid overload include diuretics and adjunctive therapy with intravenous vasodilators. Although their effects have been thoroughly assessed, diuretics cause numerous side effects [1].

Recently, vasopressin type 2 receptor antagonists have been developed. One of these drugs, tolvaptan (TLV), is available for heart failure patients with hyponatremia or symptomatic congestion [2]. The previous studies have revealed that TLV’s effects on fluid volume are associated with symptomatic improvements in hospitalized patients with worsening heart failure [2]. However, little is known about the role of TLV on fluid management after cardiovascular surgery, as few studies have assessed it [3]. The aim of this study was to identify predictive factors for responders to TLV after cardiovascular surgery.

## Methods

### Ethical considerations

This study was approved by the institutional review board of Osaka University Hospital. The application of the Osaka Cardiovascular Surgery Research Group database was approved by each affiliated hospital.

### Study participants

Among a total of 1283 patients who underwent cardiovascular surgery at three institutes in the Osaka Cardiovascular Surgery Research Group between January 2013 and May 2014, 113 patients were treated with TLV for fluid management during the postoperative period. Baseline and surgical characteristics of the patients are shown in Table 1.

**Table 1:** Baseline and surgical characteristics of all the patients

Variables	
No. of patients	113
Age (years, median)	72 ± 12
Male sex [*n* (%)]	74 (65 %)
Weight (kg, median)	58 ± 12
Surgical procedure [n (%)]	
CABG	19 (17 %)
Valve surgery	51 (45 %)
Aortic ± tricuspid	25
Mitral ± tricuspid	20
Aortic + mitral ± tricuspid	5
Tricuspid	1
CABG + valve	21 (18 %)
Others	22 (20 %)
CPB time (min, median)	172 ± 107
Ejection Fraction (%, median)	57 ± 15
Serum creatinine level (mg/dL, median)	1.1 ± 0.6
Concomitant diuretics [n (%)]	
Furosemide	97 (85 %)
Spironolactone	52 (48 %)
Carperitide	
Duration of TLV administration (days, median)	7.0 ± 5.5
Timing of TLV administration (POD, median)	2.0
Dose of TLV [n (%)] (mg)	
7.5	93 (82 %)
15	20 (18 %)

*CABG* coronary artery bypass grafting, *CPB* cardiopulmonary bypass, *POD* postoperative days, *TLV* tolvaptan

To evaluate the effect of TLV early after open-heart surgery with detailed data including hourly urine volume (UV), which is only recorded in the intensive care unit, 31 patients who received TLV at the first day after open-heart surgery using cardiopulmonary bypass (CPB) were classified as the early TLV group. Another 31 consecutive patients who were treated with the conventional diuretic therapy after open-heart surgery during the study period were identified as the control group. Baseline and surgical characteristics of patients in both the groups are shown in Table 2.

**Table 2:** Comparison of baseline and surgical characteristic between the early TLV group and the control group

Variables	Early TLV group	Control group	*p* value
No. of patients	31	31	
Age (years, median)	73 ± 13	73 ± 17	0.99
Weight (kg, median)	59 ± 14	55 ± 12	0.35
Surgical procedure [*n* (%)]			
CABG	2 (0.6 %)	0 (0 %)	0.14
Valve surgery	17 (55 %)	25 (80 %)	0.02
Aortic ± tricuspid	6	15	
Mitral ± tricuspid	9	8	
Aortic + mitral ± tricuspid	2	2	
CABG + valve	9 (29 %)	6 (20)	0.20
CPB time	221 ± 50.8	218 ± 82	0.62
Ejection fraction (%, median)	59 ± 17	66 ± 11	0.01
Serum creatinine level (mg/dL, median)	1.07 ± 0.48	0.81 ± 0.22	<0.01
Concomitant diuretics [*n* (%)]			
Furosemide	25 (80 %)	20 (64 %)	0.60
Spironolactone	7 (23 %)	10 (32 %)	0.44
Carperitide	10 (32 %)	14 (45 %)	0.38

*CABG* coronary artery bypass grafting, *CPB* cardiopulmonary bypass, *TLV* tolvaptan

To eliminate the effects of other diuretics, patients with any change in the dose of concomitant diuretics during TLV administration were excluded.

### Parameters to assess the effectiveness of TLV and study design

Two parameters were assessed as indicators of TLV effectiveness for fluid management after cardiovascular surgery. The first parameter, which was evaluated in all the patients, was change in BW (kg) during the TLV administration period: (BW before TLV administration—BW at the end of the TLV administration period). The second parameter, which was evaluated in patients in the early TLV group, was the ratio of UV before and after TLV administration (UV during the first 6 h after administration/UV during the 6 h before administration). As the peak blood concentration of TLV is obtained at around 4 h after TLV administration and urine excretion rate reaches a maximum within around 6 h after dosing in patients with heart failure [4], UV during the first 6 h after the initial TLV administration was compared with that during the 6 h before administration in the early TLV group.

Pre-operative variables included gender, age, serum creatinine level, brain-type natriuretic peptide, and cardiac function quantified using the ejection fraction (%) obtained on echocardiogram. Intraoperative fluid balance and CPB time were evaluated. During the postoperative follow-up, change in BW (BW before TLV administration—pre-operative BW), serum albumin level before TLV administration, and the dose of TLV were obtained.

In all the patients, the relationship between the perioperative variables and change in BW during TLV administration was assessed. In patients in the early TLV group, the relationship between the perioperative variables and the ratio of UV during 6 h before and 6 h after TLV administration were assessed. For the variables that showed a significant correlation with an increased UV ratio in the early TLV group, the cut-off point was evaluated using the receiver-operating characteristic (ROC) analysis for factors associated with the effectiveness of TLV.

In addition, BW change and UV during first postoperative day were compared in the early TLV group and the control group.

### Perioperative management, including administration of TLV

Open-heart surgery was performed by median sternotomy using CPB with moderate hypothermia. Our standard protocol for fluid management after cardiac surgery is: intravenous furosemide with or without carperitide infusion is generally used as the initial diuretic therapy. Furosemide infusion is usually discontinued when a patient can take oral furosemide. The administration of oral furosemide is usually started in combination with spironolactone. During the study period, the administration of TLV was considered when the standard protocol resulted in a poor response. The evidence of fluid retention (massive pleural effusion and apparent edema) could also be indications for TLV administration.

Other conventional diuretics had previously been administered at the time of TLV administration continued during TLV administration. TLV was generally given until BW returned to the pre-operative level for most patients.

### Statistical analysis

All the statistical analyses were performed with Jmp Pro 10 (SAS Institute, Cary, NC, USA). Values are mean ± standard deviation. The Mann–Whitney *U* test was used to compare continuous variables, and Fisher’s exact test was used to compare frequencies between groups. To construct categorical variables from continuous data, the cut-off point was obtained with the ROC analysis. A *p* value of <0.05 was considered statistically significant.

## Results

### TLV administration

Follow-up was completed in all 113 patients, including 31 patients who received TLV at the first day after surgery. TLV dose was 7.5 mg in 93 patients and 15 mg in 20 patients. The duration of TLV administration was 7.0 ± 5.5 days. The median timing of the initial TLV administration was 2 days after surgery. Other diuretics used concomitantly with TLV included furosemide (97 patients), spironolactone (52 patients), and carperitide (20 patients). The dose of concomitant furosemide was 40 mg (18 patients), 20 mg (72 patients), and an infusion rate of 5 mg/h in seven patients. All the patients survived surgery. Postoperative hypernatremia >150 mEq/L was seen in two patients. One patient underwent mitral valve replacement, tricuspid annuloplasty, and coronary artery bypass grafting. His pre-operative cardiac function was preserved with an ejection fraction of 61 %, and renal function was mildly deteriorated with a serum creatinine level of 1.24 mg/dL. He was started on 7.5 mg TLV at 6 days post-operation because of persisted pleural effusion. Forty milligrams furosemide and 50 mg spironolactone were concomitantly administered. Three days after starting administration, his serum Na level rose to 152 mEq/L from a baseline level of 144 mEq/L without any symptoms. TLV administration ceased and his serum Na level improved within 2 days. The other patient underwent aortic valve replacement and coronary artery bypass grafting. His pre-operative cardiac function was preserved with an ejection fraction of 70 %, and renal function was favorable with a serum creatinine level of 0.86 mg/dL. He suffered from massive pulmonary bleeding immediately after weaning from CPB. He had been managed with extracorporeal membrane oxygenation for 11 days postoperatively because of a deteriorating respiratory condition. Thirty milligrams TLV was initiated at 5 days post-operation in an attempt to treat the edematous state of his lungs. Furosemide infusion at a rate of 5 mg/h was concomitantly conducted. Ten days after starting administration, his serum Na level rose to 153 mEq/L from a baseline level of 136 mEq/L. Because the patient was intubated and mildly sedated, the symptoms of hypernatremia could not be precisely evaluated. After the cessation of TLV, his Na level improved within 2 days. In all the patients, the mean baseline Na level was 141 mEq/L, and the mean peak level after TLV administration was 142 mEq/L (*p* ≤ 0.001).

A postoperative serum creatinine level >2.0 mg/dL was seen in 12 patients. Among them, nine showed pre-operative renal dysfunction with a creatinine level >2.0 mg/dL. In all the patients, creatinine levels returned to pre-operative levels by 7 days after the initial administration of TLV.

### Change in BW during TLV administration period

**Fig. 1:**
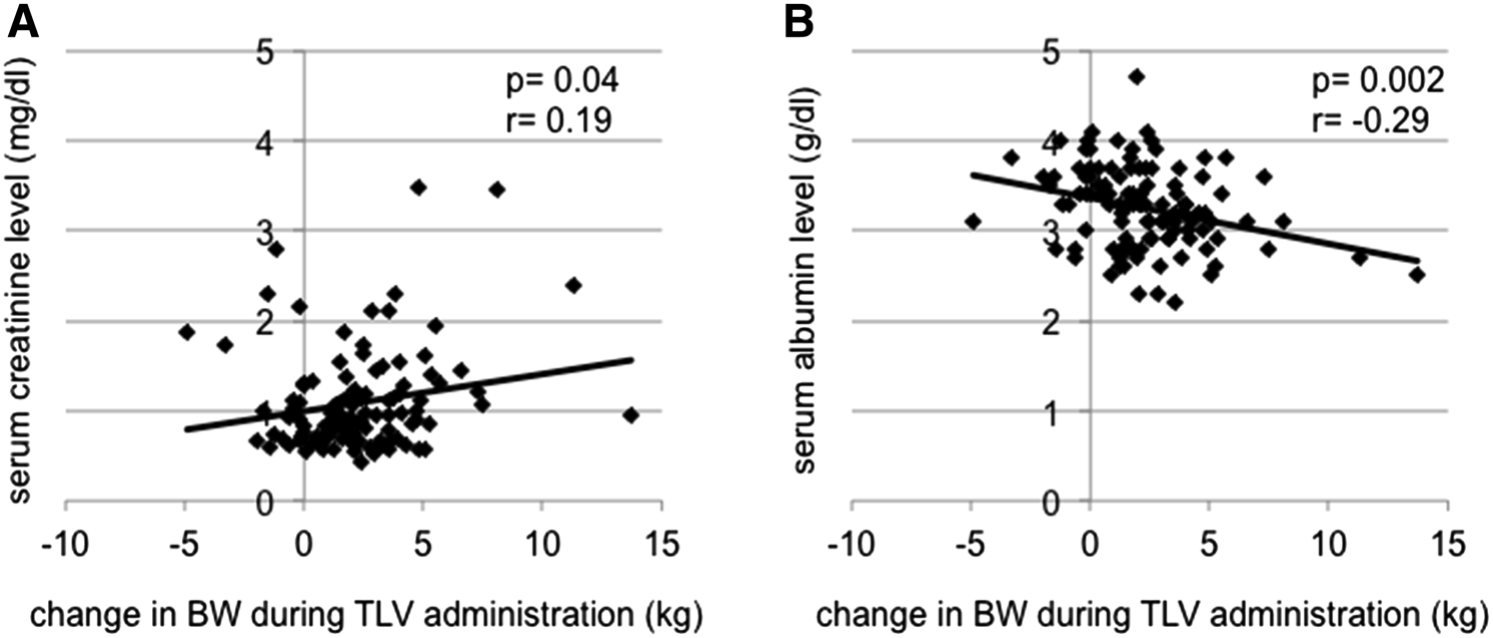
**a** Correlation between pre-operative serum creatinine level and change of BW during TLV administration. **b** Correlation between serum albumin level before TLV administration and change of BW during TLV administration. *BW* bodyweight, *TLV* tolvaptan

In all the patients, change in BW during the TLV administration period correlated significantly with serum creatinine level (*r* = 0.19, *p* = 0.04) (Fig. 1a) and serum albumin level before TLV administration (*r* = −0.29, *p* = 0.002) (Fig. 1b). There were no significant correlations between change in BW and gender, age, brain-type natriuretic peptide, left ventricular ejection fraction, intraoperative fluid balance, or intraoperative CPB time. There was no significant difference in change in BW according to dose of TLV (*p* = 0.83).

### Ratio of UV before and after the initial TLV administration

**Fig. 2:**
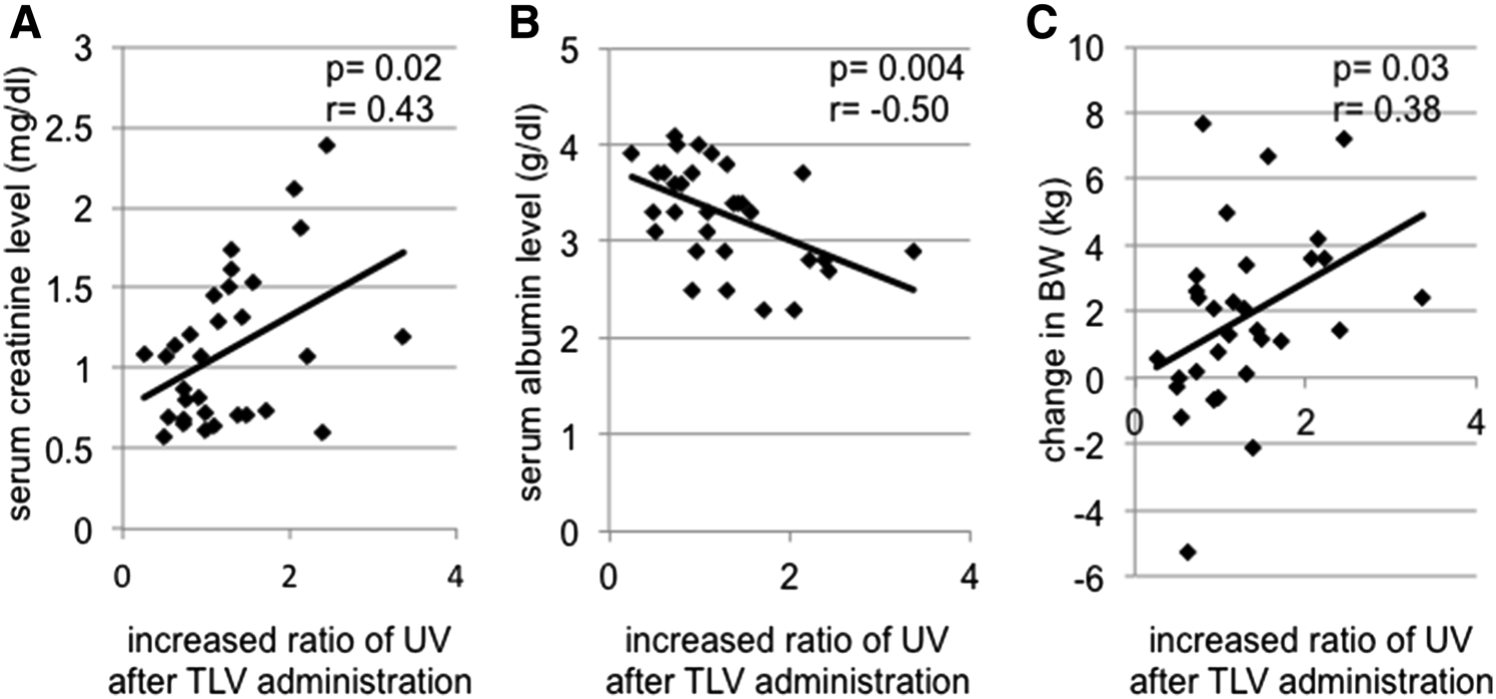
**a** Correlation between pre-operative serum creatinine level and increase ratio of UV after TLV administration. **b** Correlation between serum albumin level before TLV administration and increase ratio of UV after TLV administration. **c** Correlation between change of BW before TLV administration and increase ratio of UV after tolvaptan administration. *TLV* tolvaptan, *UV* urine volume, *BW* bodyweight

In the early TLV group, the ratio of UV 6 h after to 6 h before the initial TLV administration significantly correlated with the pre-operative serum creatinine level (*r* = 0.43, *p* = 0.02) (Fig. 2a), the serum albumin level before TLV administration (*r* = −0.50, *p* = 0.004) (Fig. 2b), and change in BW (*r* = 0.38, *p* = 0.03) (Fig. 2c). There was no significant difference in the ratio of UV 6 h after to 6 h before the initial TLV administration according to the dose of TLV in the early TLV group (*p* = 0.79).

**Fig. 3:**
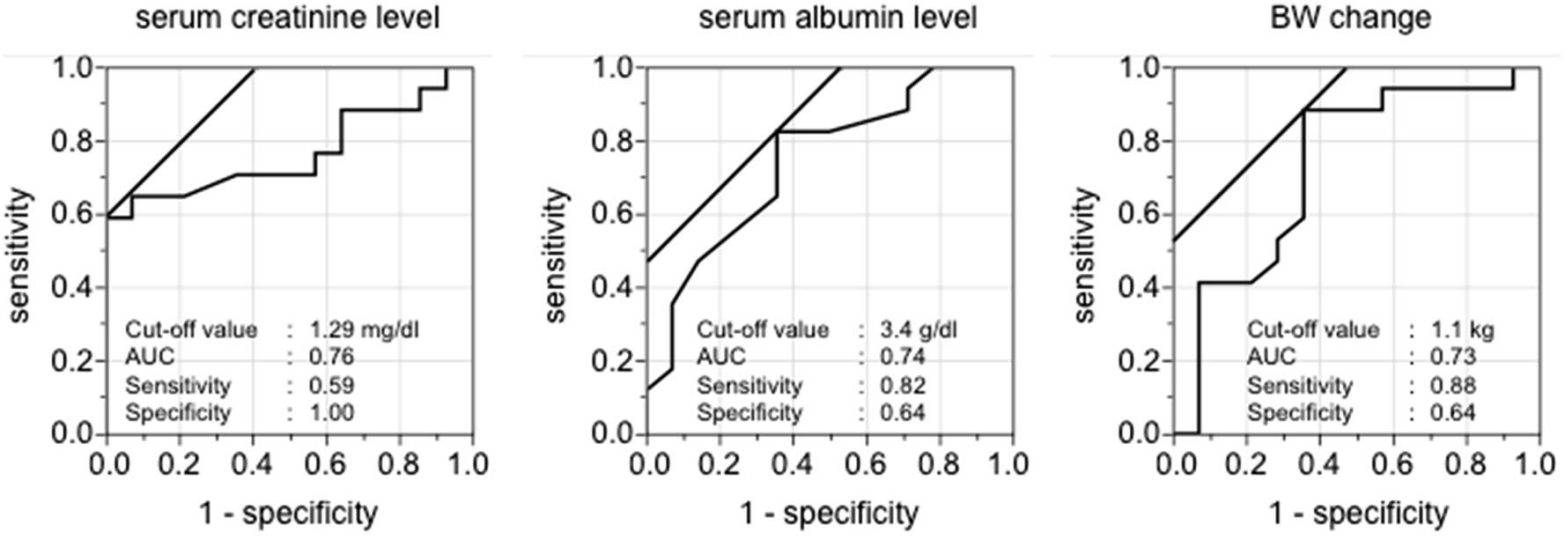
ROC analysis for patients in the early TLV group. *TLV* tolvaptan, *AUC* area under the curve, *BW* bodyweight

**Fig. 4:**
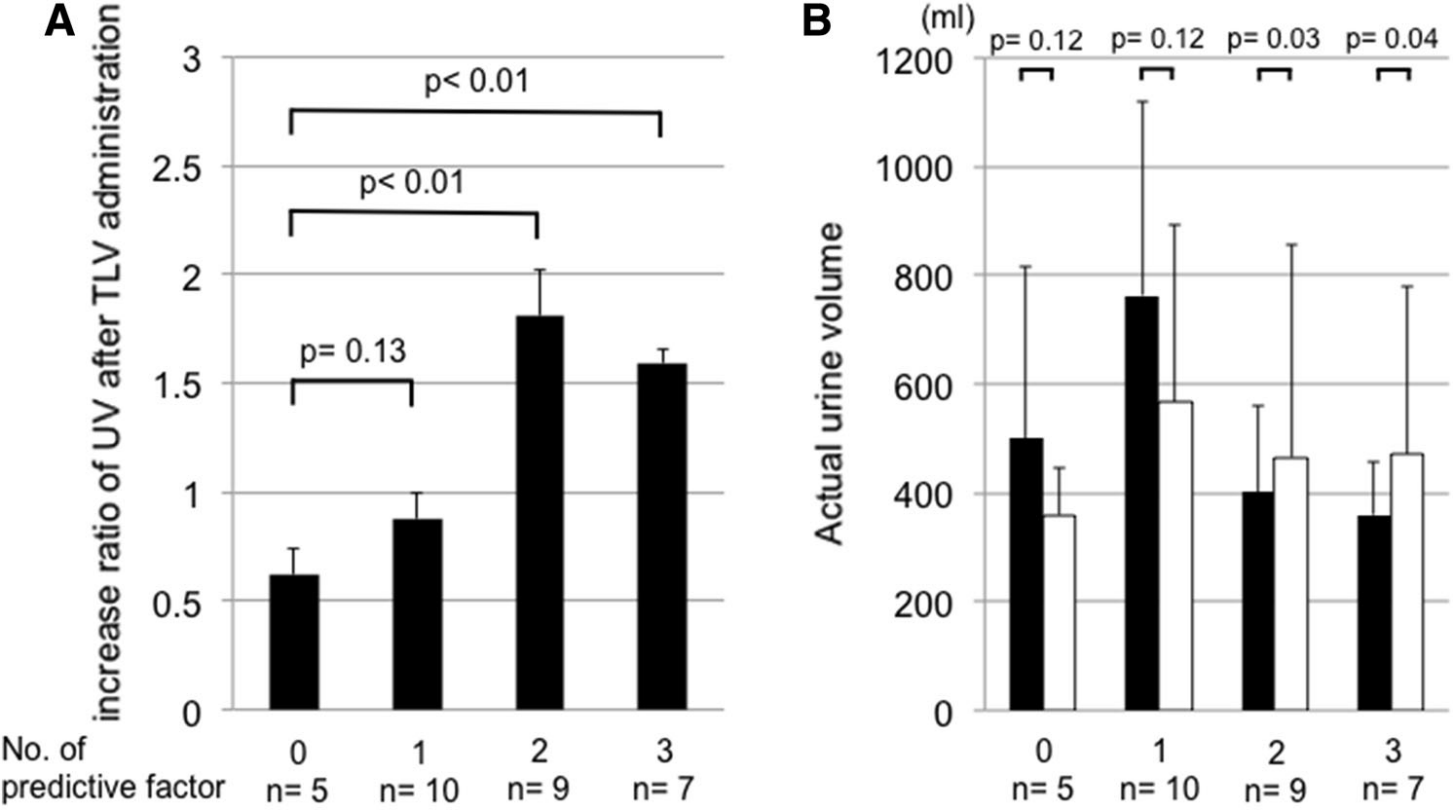
**a** Increase ratio of UV after TLV administration according to satisfied predictive factors. The effect of TLV (ratio of UV before and after TLV administration) was significantly higher in groups 3 and 4 than that in group 1. *TLV* tolvaptan, *UV* urine volume. **b** Actual urine volume during 6 h before and 6 h after TLV administration according to satisfied predictive factors. *Black bar* represents urine volume during 6 h before TLV administration and white bar represents urine volume 6 h after TLV administration. *TLV* tolvaptan, *UV* urine volume

Cut-off values were calculated for serum creatinine (1.29 mg/dL), serum albumin (3.4 g/dL), and change in BW (1.1 kg) by the ROC analysis for patients in the early TLV group (Fig. 3). Considering these cut-off levels as predictive factors for the response to TLV administration, patients in the early TLV group were divided into four groups according to the number of satisfied predictive factors (Group 1: no factors, Group 2: one factor, Group 3: two factors, and Group 4: three factors). The effect of TLV (UV after/UV before TLV administration) was significantly greater in Groups 3 and 4 compared with Group 1 (Fig. 4a). Actual UV for 6 h before and 6 h after TLV administration in each group is shown in Fig. 4b.

Among 31 patients in the early TLV group, the information of UV/h at the second postoperative day was available for 12 patients. The ratio of UV 6 h before and 6 h after TLV administration at the second postoperative day (mean 1.2 ± 0.49) showed a significant positive correlation with that at the first postoperative day (mean 1.3 ± 0.61) (*r* = 0.63, *p* = 0.03).

### Comparison of BW change and UV in the early TLV group and the control group

Patients in the early TLV group showed a lower ejection fraction (*p* = 0.01) and a higher serum creatinine level (*p* ≤ 0.01) than patients in the control group (Table 2). Mean BW change and UV at the first postoperative day were 0.56 kg and 2325 ± 832 mL, respectively, in the early TLV group and 0.16 kg and 2137 ± 1010 mL, respectively, in the control group. No significant differences were detected for both parameters (*p* = 0.49 and *p* = 0.31, respectively).

## Discussion

We found that the pre-operative serum creatinine level, the serum albumin level before TLV administration, and change in BW were predictive factors for responders of TLV after cardiovascular surgery. Patients in the early TLV group showed similar UV and change in BW when compared with the control group, despite having a lower baseline ejection fraction and a higher serum creatinine level.

Since TLV has a different mechanism of diuresis than the conventional therapeutic modalities, TLV can have a great impact on postoperative management. It is important to clarify the ideal candidate for TLV administration. In the previous studies of heart failure patients, a decrease in urine osmolality of more than 26 % from a baseline >352 mOsm/L for the first 4–6 h predicted a response to TLV [5]. While patients with renal dysfunction are reported to show relatively smaller increases in UV after TLV administration [5], patients in the current study following cardiovascular surgery showed the opposite result. Decreased renal function often contributes to increased intraoperative fluid balance and a change in BW, which result in interstitial edema after cardiovascular surgery. Costello-Boerrigter et al. reported that renal blood flow and glomerular filtration rate are maintained after TLV administration [6]. In addition, TLV is expected to exhibit a diuretic effect without activating the renin-angiotensin system [7, 8]. In the current study, the effect of TLV was greater in patients with a higher serum creatinine level, possibly because of the severely edematous state of patients with decreased renal function and the renoprotective effect of TLV.

After cardiovascular surgery, hemodilution and systemic inflammatory responses cause increased capillary permeability and decreased colloid osmotic pressure, which result in interstitial edema. Hypoalbuminemia results from the progression of protein catabolism after cardiovascular surgery. Because albumin is the molecule mainly responsible for intravascular osmotic pressure, the serum albumin level is considered an indicator of the edematous state. Therefore, hypoalbuminemia may predict the response to TLV, as demonstrated in the current study.

No significant increase in UV after TLV administration was seen in patients with preserved renal function and non-hypoalbminemia (Groups 1 and 2). This may be because of sufficient UV before TLV administration in these patients. In patients in the early TLV group, the effect of TLV after the initial administration was similar to that after the second administration. During the early postoperative period after cardiovascular surgery, the effect of TLV seems to be consistent. The effect of TLV is usually dose-dependent [4], yet there was no significant difference according to the dose of TLV in the current study. In terms of the effect of TLV in fluid management after cardiovascular surgery, the number of satisfied predictors might be more important than the dose of TLV.

This study had some limitations. The study was conducted retrospectively and had a limited number of patients. Because the current use of TLV is approved only for situations, where other conventional diuretics are concomitantly used, bias stemming from the effect of other concomitant diuretics could not be totally eliminated. The dose and duration of TLV treatment were not standardized, as they were determined by each physician after considering the patient’s perioperative status and background. Considering the mechanism of action of TLV, the plasma concentration of vasopressin and urine osmolality before TLV administration might have predictive potential for responders of TLV; however, we have no data on these variables.

## Conclusion

TLV might have a beneficial effect on fluid management in patients after cardiovascular surgery, especially in those with decreased renal function, increased BW, and hypoalbuminemia. Although we were able to identify predictive factors for responders to TLV after cardiovascular surgery in the current study, further studies in which TLV is used without any other diuretics for postoperative management after cardiovascular surgery are needed.
